# Beg, Borrow and Steal: Three Aspects of Horizontal Gene Transfer in the Protozoan Parasite, *Cryptosporidium parvum*

**DOI:** 10.1371/journal.ppat.1005429

**Published:** 2016-03-03

**Authors:** Adam Sateriale, Boris Striepen

**Affiliations:** 1 Center for Tropical and Emerging Global Diseases, University of Georgia, Paul D. Coverdell Center, Athens, Georgia, United States of America; 2 Department of Cellular Biology, University of Georgia, Paul D. Coverdell Center, Athens, Georgia, United States of America; University of Wisconsin Medical School, UNITED STATES

## What Is *Cryptosporidium*?

*Cryptosporidium* infection, or cryptosporidiosis, was first described in mice by Ernest Tyzzer in 1907, yet infection of humans went unrecognized until 1976 [1,2]. The emergence of HIV brought human cryptosporidiosis to the fore, in the 1980s, as a chronic and life threatening opportunistic infection. Most recently, a series of epidemiological studies have revealed the truly ubiquitous nature of cryptosporidiosis and its massive impact on global public health. Young children are highly susceptible to cryptosporidiosis, and in one of the recent epidemiological surveys—the Global Enteric Multicentre Study (GEMS)—cryptosporidiosis was found to be a major contributor to severe diarrheal disease in children under the age of two [3]. Severe cryptosporidiosis is closely associated with mortality, yet children who survive infections, even asymptomatic infections, can suffer from lasting growth and developmental defects [3–5]. *Cryptosporidium* parasites are environmentally hardy, withstanding common water treatments such as chlorination, and thus remain a major cause of waterborne outbreaks in industrialized countries as well [2].

*Cryptosporidium* belong to the eukaryotic phylum Apicomplexa, distant relatives of the parasites that cause malaria and toxoplasmosis. Although many species and strains of *Cryptosporidium* have been reported to infect humans, the majority of infections are caused by *Cryptosporidium hominis* and *Cryptosporidium parvum*. *C*. *hominis* and *C*. *parvum* are closely related, differing at the nucleotide level by 3%–5% [6,7]. Both of their genomes are small (approximately 9 Mb), adenosine- and thymidine-rich (approximately 70%), and contain a relatively large proportion of genes acquired from other organisms by horizontal gene transfers (HGTs) [8]. HGTs have clearly had a significant influence on the evolution of *Cryptosporidium*, and here we will highlight just three: metabolism in an anaerobic environment, salvage of nucleotides from the infected host, and one HGT that may allow *Cryptosporidium* to evade host immunity.

## Anaerobic Metabolism

**Fig 1:**
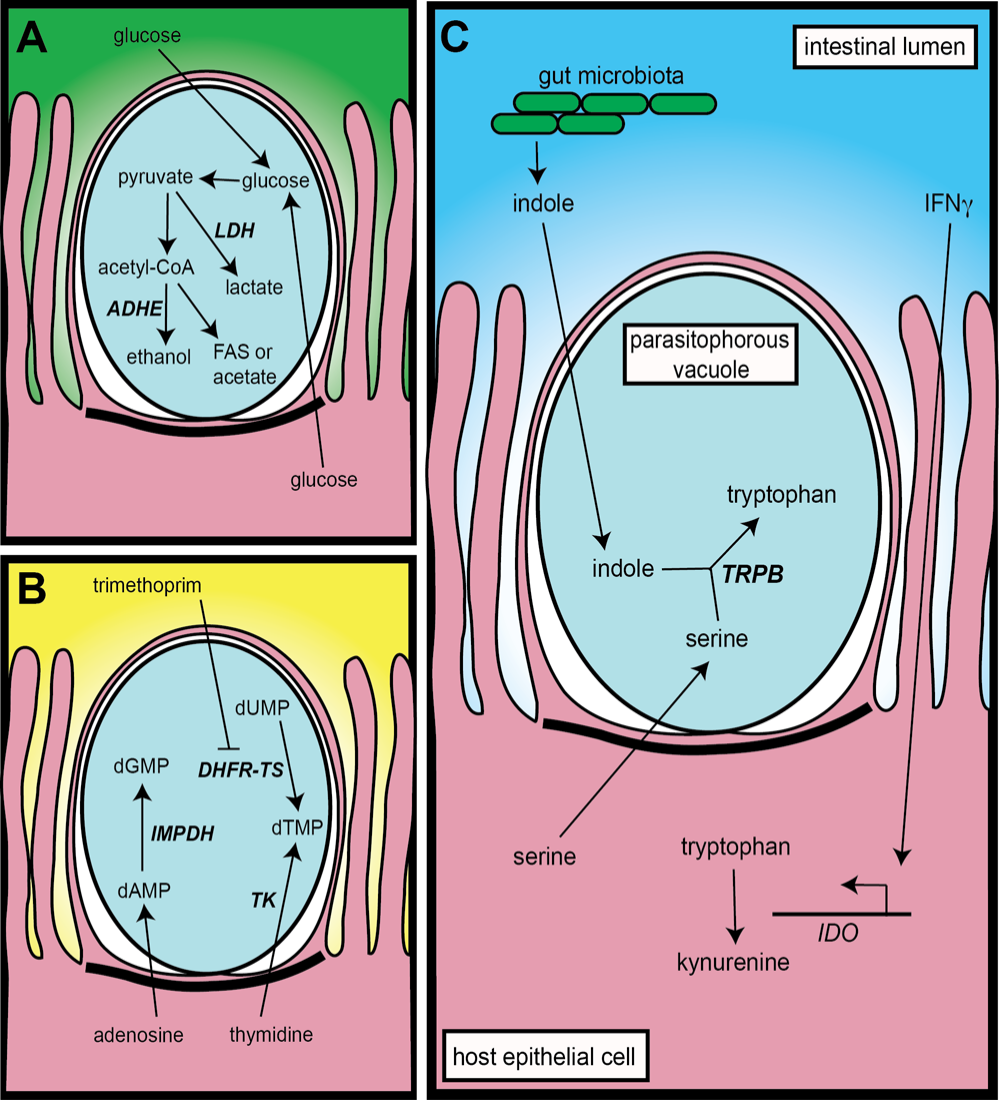
Horizontally transferred genes and their proposed functions in *Cryptosporidium* biology. **A. Anaerobic metabolism**. *Cryptosporidium* can regenerate oxidized NAD either directly from pyruvate via lactate dehydrogenase (LDH, *cgd7_480*) or from acetyl-CoA via a bifunctional alcohol dehydrogenase (ADHE, *cgd8_1720*). B. Nucleotide salvage. *Cryptosporidium* relies on inosine 5′ monophosphate dehydrogenase (IMPDH) to convert deoxyadenosine monophosphate (dAMP) to guanosine monophosphate (dGMP) within the parasitophorous vacuole. Dihydrofolate reductase-thymidylate synthase (DHFR-TS, *cgd4_4460*) and thymidine kinase (TK, *cgd5_4440*) can both produce deoxythymidine monophospohate (dTMP); however, the presence of TK allows *Cryptosporidium* to survive treatment with dihydrofolate reductase (DHFR) inhibitors such as pyrimethamine and trimethoprim. C. Immune evasion. Interferon gamma (IFNγ) induces the production of indoleamine 2,3-deoxygenase (IDO) within infected host cells, which converts host tryptophan to kynurenine and starves intracellular organisms. *Cryptosporidium* has a horizontally acquired tryptophan synthase B (TrpB, *cgd5_4560*), possibly to evade this starvation. TrpB is capable of synthesizing tryptophan from exogenous serine and indole produced by gut microbiota.

*Cryptosporidium* species have a metabolism properly suited to the anaerobic environment of the host intestine. The majority of cellular energy is thought to be derived from glycolysis, the oxygen-independent conversion of glucose to pyruvate. Glycolysis produces cellular ATP, but also produces the reduced form of nicotinamide adenine dinucleotide (NAD(P)H). This important coenzyme must then be regenerated back into its oxidized form (NAD(P)) to allow glycolysis to proceed, but in the host intestine, this has to be accomplished in the absence of oxygen. In order to adapt to the anaerobic environment of the intestine, *Cryptosporidium* horizontally acquired two genes to regenerate oxidized nicotinamide adenine dinucleotide (NAD): alcohol dehydrogenase and lactate dehydrogenase (Fig 1A). Bacterial-type alcohol dehydrogenases are not uncommon in intestinal parasites, and it appears that there have been multiple independent acquisitions. *Giardia lamblia* and *Entamoeba histolytica*, two phylogenetically distant eukaryotes, also have alcohol dehydrogenases of bacterial lineage [9,10]. *Cryptosporidium* has a bifunctional alcohol dehydrogenase (ADHE), akin to the EhADHE of *E*. *histolytica*, which first converts acetyl CoA (not pyruvate) to acetaldehyde, then acetaldehyde to ethanol. This horizontal transfer likely occurred from an ancestral gut bacterium, and BLAST alignments show half of the amino acid sequence to be identical to the oxygen-independent alcohol dehydrogenases of the *Clostridium* genus. *Cryptosporidium* can also regenerate NAD directly from pyruvate through the activity of a lactate dehydrogenase (LDH). Phylogenetic evidence supports the hypothesis that an ancestral member of the *Apicomplexan* family of parasites first acquired this gene from a proteobacteria prior to familial separation [8,11]. Post-separation, this gene duplicated within *Cryptosporidium*, and the function of each new gene diverged, leading to a lactate dehydrogenase and a malate dehydrogenase that now sit adjacent to each other within the parasite genome. Recent work has demonstrated that the localization of lactate dehydrogenase is remarkably dynamic during *Cryptosporidium* infection. LDH exists in the cytosol of extracellular parasites, then relocates to the membrane of the parasitophorous vacuole following infection [12]. The functional significance of this interesting localization is yet to be discovered.

## Nucleotide Salvage

*Cryptosporidium* nucleotide biosynthesis is largely shaped by genetic reduction as they have lost (or, perhaps more aptly stated, “discarded”) the ability to synthesize nucleotides de novo [13]. *Cryptosporidium* relies on its host through the salvage of both purines and pyrimidines, and two horizontally transferred genes aid in these processes: inosine 5′ monophosphate dehydrogenase (IMPDH) and thymidine kinase (TK) (Fig 1B). Both genes appear to be acquired from ancestral proteobacteria, but in separate transfer events [8]. IMPDH converts inosine monophosphate to xanthosine monophosphate, an important step in the biosynthesis of guanosine monophosphate. *Cryptosporidium* lacks other recognized pathways to produce guanine monophosphate; therefore, IMPDH function appears to be essential for DNA replication and survival [13]. Humans have an IMPDH that is very different from the bacterial enzyme, which makes it an attractive target for drug development. IMPDH inhibitors have been shown to be safe and effective against *C*. *parvum* using the interleukin 12 deficient mouse model of infection [14]. In contrast to IMPDH, the horizontally acquired TK gene is not essential to *Cryptosporidium* survival. Transgenic *Cryptosporidium parvum* parasites that lack thymidine kinase have no measurable growth defects and produce robust infections in mice [15]. However, these TK-deficient parasites are highly susceptible to dihydrofolate reductase (DHFR) inhibitors. This class of drugs (including trimethoprim and pyrimethamine) has been a mainstay in the treatment of malaria and toxoplasmosis but is ineffective against *Cryptosporidium*. Without thymidine kinase, however, trimethoprim and pyrimethamine cure human intestinal epithelial cells of *C*. *parvum* infection, and thus the horizontal acquisition of TK appears to generate the redundancy responsible for *Cryptosporidium’s* drug resistance [15].

## Immune Evasion?

The pro-inflammatory cytokine interferon gamma (IFNγ) has a central role in the host immune response to *Cryptosporidium* [16,17]. Individuals with low or defective IFNγ are susceptible to chronic infections similar to those seen in patients with low CD4 counts [18]. Similarly, IFNγ knockout mice are readily infected with *C*. *parvum*, whereas the parental mouse strain is resistant to infection [17]. While the effects of IFNγ are pleiotropic, one important consequence for intracellular parasites is the up-regulation of host indoleamine 2,3-deoxygenase (IDO). IDO rapidly converts cellular tryptophan to kynurenine, effectively starving intracellular invaders of this important amino acid [19]. However, *Cryptosporidium* have a horizontal acquisition that may specifically circumvent tryptophan starvation—tryptophan synthase B (TrpB), another enzyme encoded by a gene that appears to be of proteobacterial origin (Fig 1C) [8]. TrpB is present in many intracellular bacterial pathogens, notably S*almonella*, *Chlamydia*, and *Mycobacterium* species. In the face of IFNγ-induced tryptophan starvation, TrpB can produce tryptophan from the condensation of serine with the aromatic compound indole, which is produced in large amounts by the host’s resident microbiota. *Chlamydia trachomatis* has been shown to use TrpB to specifically escape IFNγ-induced starvation, and strains with disrupted tryptophan synthesis are virulence-attenuated [20]. If *Cryptosporidium* TrpB acts in an analogous fashion, disruption of the gene should yield attenuated parasites with diminished ability to withstand IFNγ. Alternatively, *Cryptosporidium* TrpB may have a unique function independent of IFNγ; bacterial communities use indole as a signaling molecule with wide-ranging effects on drug resistance, virulence, and the formation of biofilms [21]. *Cryptosporidium* may be listening in on this complex bacterial conversation to derive developmental clues. Consistent with a potential link to the microbiota, TrpB is found in *Cryptosporidium* species that infect the intestine but not in species that infect the stomach or airways. These hypotheses on the function of TrpB and other horizontally transferred genes need to be put to a rigorous test.

## The Utility of Studying HGTs

Now that we appreciate the enormous impact that cryptosporidiosis has on public health, the development of therapeutic and preventive interventions are a high priority. The standard of care for cryptosporidiosis is the drug nitazoxanide, which shows little efficacy in malnourished children and is comparable to placebo in immunocompromised patients (reviewed in [22]). There are no vaccines in development or trial. Genetic manipulation of the parasite has recently become possible, and this technology will undoubtedly aid in the drug-discovery process [15]. Parasites with engineered reporters will facilitate high throughput drug screening, and targeted mutations can validate putative drug targets. Genetic manipulation will also further our understanding of basic *Cryptosporidium* biology, and a logical place to focus is the function of HGTs. HGTs in *Cryptosporidium* are mostly prokaryotic in origin, which may simplify drug development, and of great enough importance to have become fixed within the parasite genome. These qualities make HGTs attractive targets of therapeutic intervention, and they may also represent opportunities for parasite attenuation in the context of vaccine research.
